# Facial Nerve Revascularization Strategies in Facial Restoration

**DOI:** 10.1097/GOX.0000000000004038

**Published:** 2022-01-13

**Authors:** Ankur Khajuria, Brian Bisase, Paul Norris, Jag Dhanda, Isao Koshima, Charles Nduka, Ruben Y. Kannan

**Affiliations:** From the * Kellogg College, University of Oxford, Oxford, UK; † Department of Surgery & Cancer, Imperial College London, London, UK; ‡ Department of Plastic Surgery, Queen Victoria Hospital, East Grinstead, UK; § International Centre of Lymphoedema (ICL), Hiroshima University, Japan.

## Abstract

Facial transplants represent the current exemplar in the reconstruction of severely damaged faces, whereas conventional free flap reconstruction has its limitations in restoring both function and surface cover.

**Methods:**

In a retrospective study over 6 years (2014–2020), 5 cases (n = 5) of vascularized nerve flaps (VNFs) were performed by our team. These involved three acute and two late reconstructions. The mean age was 41 years with a maximum of 6-year follow-up. To objectify the different permutations and combinations, we categorized composite, chimeric, and hybrid VNFs into types I, IIa-c, and III, each with a unique characteristic. Postoperative function was evaluated using the validated Sunnybrook and Terzis scores for facial nerve palsy; masticatory function was assessed using dental impression studies.

**Results:**

There was a 100% flap survival rate, with no instances of flap necrosis and only one complication: hematoma at 24 hours postoperative. Sunnybrook and Terzis scores showed a statistically significant improvement postoperatively, indicating both improved repose and facial expressions (paired student *t* test, *P* < 0.05). Given that each VNF was specifically customized for a particular patient, each type of VNF in this cohort was unique, thereby illustrating each type succinctly.

**Conclusions:**

VNFs are separate entities from standard free flaps, as they require extensive preoperative planning to allow the deconstructing of composite blocks of tissue into separate vascularized entities and amalgamating them into a new conglomerate. This allows VNFs to fill a niche area in facial reconstructive surgery between face transplants and conventional free tissue transfers, with enormous potential.

**Takeaways**

**Question:** Can vascularized nerve flaps serve as an alternative to allogeneic transplants in facial restorative surgery?

**Findings:** One-hundred percent flap survival rate and statistically significant improvement in Sunnybrook and Terzis scores were found.

**Meaning:** Vascularized nerve flaps can fill a niche area in facial reconstructive surgery between face transplants and conventional free tissue transfers.

## INTRODUCTION

Large facial defects present a challenge to the reconstructive surgeon, as they require restoration of contour, function, and movement. In current times, the tendency is to veer toward composite tissue allo-transplantation,^1^ but the need for long-term immunosuppression is becoming increasing unpalatable, particularly given the recurrent outbreaks of viral pandemics of late.^2^ We ask the question as to whether there is an alternative to allogeneic transplants in facial restorative surgery.

Vascularized nerve flaps (VNFs) are an advanced version of composite and chimeric flaps and represent excellent options that provide the opportunity to successfully restore all four components of the reconstructive pyramid.^3^ Chimeric flaps^4,5^ present an invaluable instrument in the armamentarium of the plastic surgeon, as they can provide three-dimensional reconstruction, but with VNFs, we can go a step further by seamlessly restoring function in addition. These are more than just chimeric free flaps but with meticulous planning and lateral thinking, can be transformed into very complex autologous tissue transplants, which can rival their allogeneic counterparts.

VNFs can satisfy all four components of the reconstructive pyramid and with relation to facial reanimation and masticatory function, provide this either in the form of vascularized nerve grafts (VNGs) or as a vascularized tissue bed (flap) on which nerve regeneration can be sustained in a nonvascularized nerve graft. In this article, we seek to ascertain the role of VNFs in restoring facial functions such as eye closure, speech, swallowing, and mastication and classify them into different subtypes for clinical use.

## METHODS

In a retrospective study over a 6-year period (2014–2020), our team at the Queen Victoria Hospital, East Grinstead, performed five VNFs (n = 5). There was a female-to-male ratio of 3:2 and a mean age of 41 years (range: 23–56). Two cases were performed in the acute setting following radical parotidectomies, one for a severe case of necrotizing fasciitis of the face and neck in an ASA 4 patient and another two cases for (1) a delayed presentation of complete right-sided facial paralysis, following a radical parotidectomy, neck dissection and adjuvant radiotherapy with a failed Permacol static sling procedure, at over 6 months following surgery and (2) a patient with cranial polyneuropathy following excision of a cerebellar tumor as a child, resulting in facial deformity and paralysis.

**Fig. 1.**
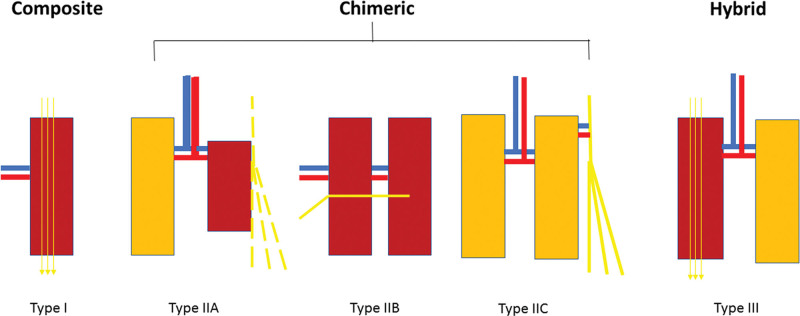
Classification for VNFs wherein type I refers to composite vascularized nerve grafts, type IIa is a chimeric flap that provides a vascular bed to a devascularized facial nerve and/or its branches, type IIb is a chimeric flap with a VNG subunit, type IIc is a flap with an independent vascularized muscle graft component, and type III is a hybrid of composite/chimeric vascularized nerve grafts and vascularized muscle grafts either as in-series (sequential) free tissue transplants or in-parallel.

The cases in this cohort were reconstructed with a customized VNF for three tier II and two tier III defects^3^ respectively. This is over and above the tier I flaps, conventionally used bar concurrent neurotization. The VNFs were classified into two major types, namely, composite (type I), chimeric (type II), and hybrid (type III) as follows. Type I VNFs include the vastus lateralis vascularized motor nerve graft (VL-MVNG), whereas chimeric or type II VNFs composed of the following subtypes: type IIa, a nonvascularized nerve-sustaining component, which serves as a vascularized tissue bed on which conventional nerve repairs or grafts can survive; type IIb, flap with an independent VNG component; and type IIc, flap with an independent vascularized muscle graft component. Type III is a hybrid of composite/chimeric vascularized nerve grafts and vascularized muscle grafts either as in-series (sequential) free tissue transplants or in-parallel.^5^ This classification system is shown in Table 1 and graphically illustrated in Figure 1.

**Table 1.** VNFs Classification System (A Restorative Template on which to Approach Complex Facial Defects)

VNFS	Description	Tiers Provided
Type I	Simple VNG	Movement
Type II	Composite VNFs with the VNG within	Volume, movement
Type IIIa	Chimeric VNFs with NVNS component, perfusing Vascularized nerve repairs/grafts	Volume, movement
Type IIIb	Chimeric VNFs with an independent VNG segment	Surface, volume, movement
Type IIIb	Chimeric VNFs with an independent VNG segment	Surface, volume, movement
Type IIIc	Chimeric VNFs with an independent VMG segment	Volume, movement
Type IV	Hybrid VNFs with both independent segments of VNGs and VMGs, either in-series (sequential) or in parallel. These consist of both composite and chimeric subunits.	Surface, volume, movement

Preoperative planning involved magnetic resonance imaging, magnetic resonance angiography, and Doppler flow studies. Postoperative facial function was assessed using the validated Sunnybrook and Terzis scores for facial nerve palsies, whereas masticatory function was evaluated using a dental bite impression and clinical assessment.

## RESULTS

There was a 100% flap survival rate with one hematoma as a complication in this study cohort. The facial artery was the donor inflow vessel in 75% of the cases and the superior thyroid artery, in the remaining 25%. The common facial vein was the outflow vein in all cases. The nerves chosen to restore oro-facial function were the ipsilateral masseteric nerve (n = 3), ipsilateral facial nerve (n = 2), and the contralateral facial nerve (n = 1). In terms of the types of VNFs performed, there was one case each of types I, IIa, IIb, IIc, and III, respectively. Orofacial function was achieved in all cases using VNFs, with aesthetic restoration in 80% of cases. One case (case 4) currently requires secondary revision procedures, which have so far been delayed because of poorly controlled diabetes. The overall summary of the cases is shown in Table 2, and specific details of selected cases to illustrate VNFs have been discussed.

**Table 2.** Overall Results of the VNFs Performed within this Cohort

Case	VNF Type	Procedure	Tier	Outcome
1	I	VL-MVNG	II	Normal smile and eye closure but no eyebrow elevation
2	IIb	Multi-vector gracilis^6^	II	Near normal smile with excellent repose
3	IIc	SCIP-LFNG VNG	III	Good eye closure, closed mouth smile, and lip puckering
4	III	VL-MNVG/FFMT+ ALT	III	Patient survived. Good eye closure and normal mastication
5	IIa	ALT-RF	II	Full preservation of VII and complete recovery of frontalis

VL-MVNG, composite vastus lateralis-motor vascularized nerve graft; SCIP-LCFN VNG, chimeric superficial circumflex iliac perforator flap with a VNG component (viz., lateral femoral cutaneous nerve); VL-MVNG/VMG + ALT, hybrid vastus lateralis motor vascularized nerve graft/free functional muscle transfer + ALT sequential component in-series.

## CASE ILLUSTRATION

### Case 1

**Fig. 2.**
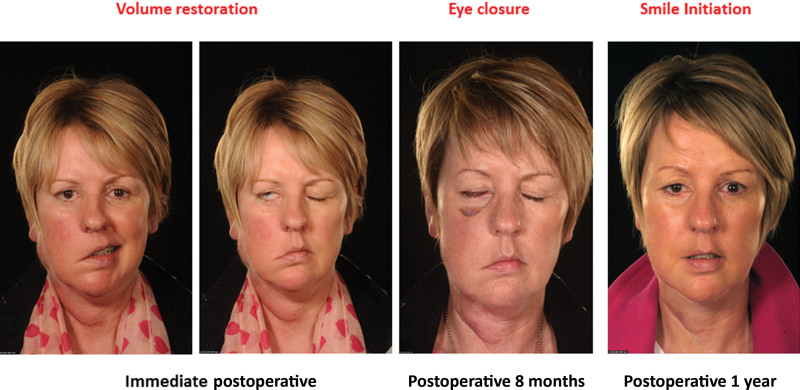
Serial photographs showing gradual improvement in facial expressions from the immediate postoperative period throughout the 5 years postoperative.

A 46-year-old woman presented with a parotid cancer, which required a radical parotidectomy and VII sacrifice with postoperative irradiation planned. At the primary procedure, a VL-MVNG (type I VNF) was used in a tier II reconstruction to restore both volume and facial expressions after surgery. Serial follow-up showed eye closure by 8 months, closed lip smile at 1 year (Fig. 2), and fully restored lip pucker, cheek blowing, and open mouth smile at 3 years postoperative. She also has excellent facial contour and normal facial repose. (See Video [online], which displays case 2 patient at 3 years postoperative, exhibiting optimal symmetry at rest, eye closure, open mouth smiling, and lip puckering.)

**Video 1:** displays the patient in case 2 at 3 years postoperative, exhibiting optimal symmetry at rest, eye closure, open mouth smiling and lip puckering.

### Case 2

**Fig. 3.**
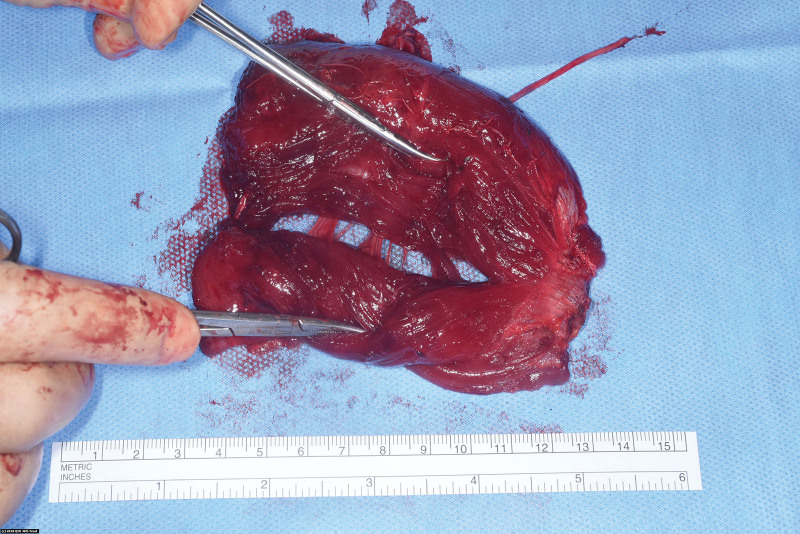
An intraoperative image of a type IIb VNF with two separate chimeric subunits of the gracilis FFMT in-series.

A 23-year-old man had a cerebellar ependydoma excised at the age of 14, leaving him with a lower motor neuron type polycranial neuropathy of the oculomotor (III) and VII nerves as well as a significant contour deformity over the left mid- and lateral hemi-face. A single-stage left-sided dual-innervated^7^ multi-vector gracilis-free functional muscle transfer or vascularized muscle graft (Fig. 3) was performed by Boahene et al.^6^ At 20 months, type IIc VNF showed excellent symmetry at rest, facial contour, and a near normal spontaneous open mouth smile with optimal smile indices.

### Case 3

**Fig. 4.**
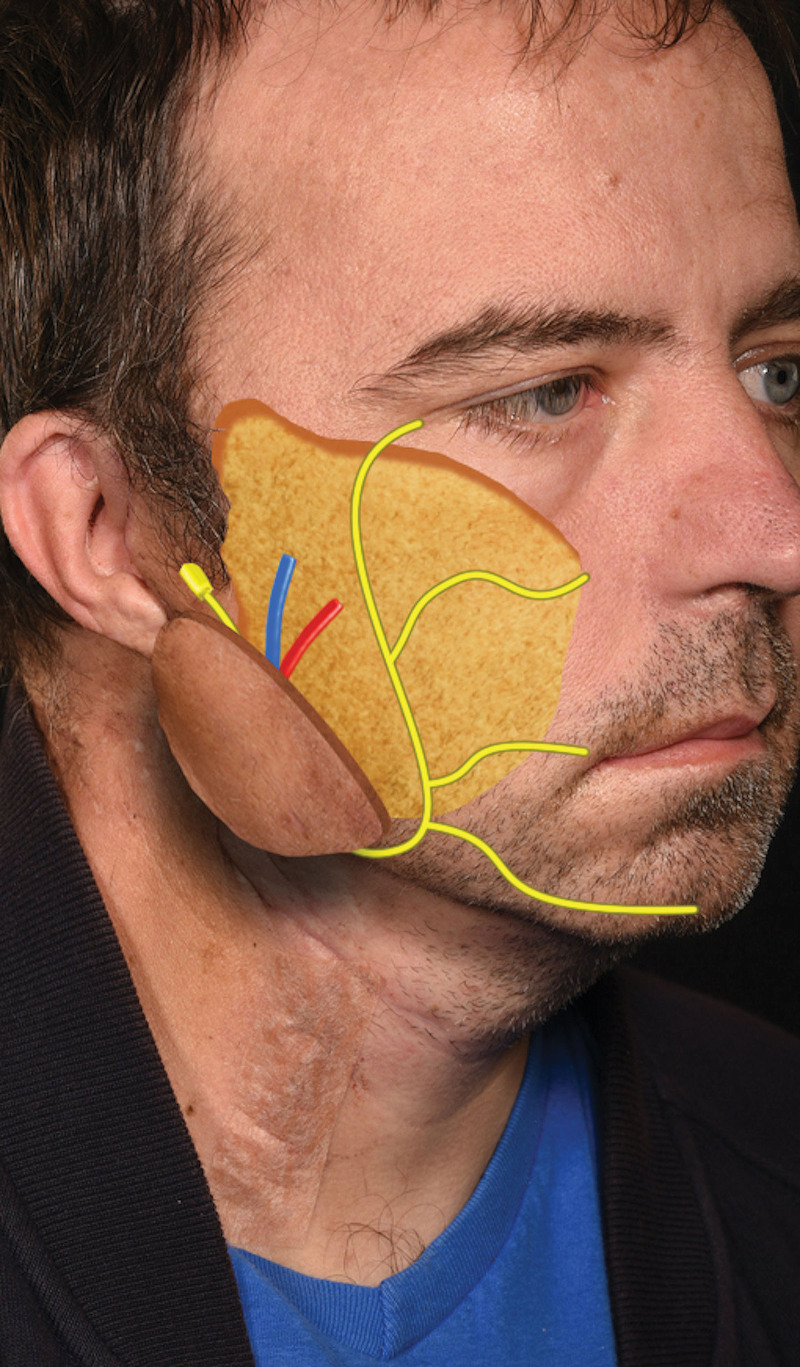
Superimposition of the type IIc VNF comprising the SCIP flap with three subunits (viz., superficial SCIP, deep SCIP, and the LCFN VNG) onto the affected right hemiface of the patient in case 3.

**Fig. 5.**
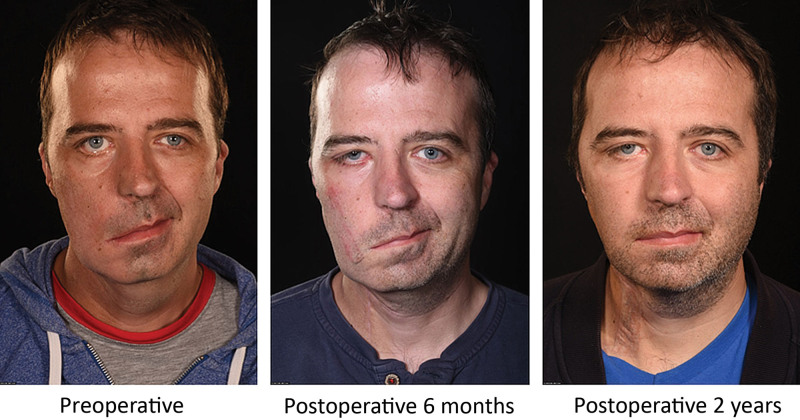
Serial photographs of case 3 patient showing gradual improvement in closed lip smiling, over a 2-year period.

**Fig. 6.**
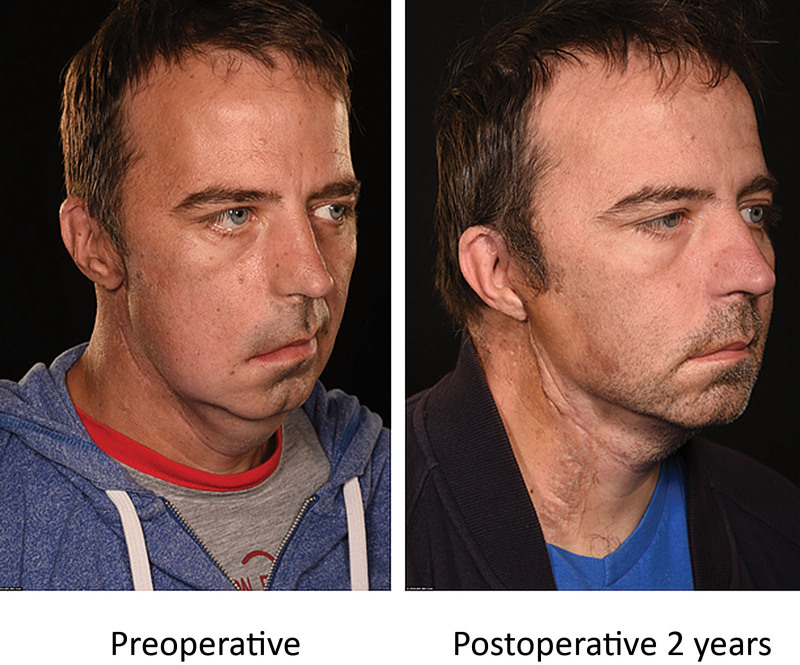
Right oblique view of case 3 patient showing the preoperative deficiency in skin and volume over the mid- and lateral face, and the restoration of mid-facial volume and and jawline definition at 2 years postoperative.

A 41-year-old man presented with severe right-sided facial pain, a lower motor neuron-type facial palsy, a significant contour deficit at 4 months postradical parotidectomy, neck dissection and radiotherapy as well as a failed Permacol sling for attempted symmetry at rest. A type IIb VNF—a chimeric superficial circumflex iliac artery with lateral circumflex femoral nerve (SCIP-LCFN) VNG—was used to restore volume, movement, and symmetry (Fig. 4). At 2 years postsurgery, the patient was able to close eyes and achieve a reasonable closed lip smile (Fig. 5). The superficial and deep SCIP components successfully recontoured his lateral and mid-face, respectively, in terms of volume fill, alongside improving jawline aesthetics, as shown in Figure 6.

In terms of facial reanimation, the mean Sunnybrook score after the lower motor neuron facial paralysis in these cases increased from a mean of 9% preoperative to 58% postoperative (paired Student *t* test, *P* = 0.014; < 0.05), whereas their Terzis smile score, which specifically measures open mouth smile, increased from a pre-op mean of 2 to 3.8, postoperative (paired student *t* test, *P* = 0.018; < 0.05). Despite the small numbers in what is otherwise a rare procedure, this was statistically significant (GraphPad PRISM, USA). In terms of masticatory function, the patient in case 4 exhibited no evidence of occlusion or inability to chew despite having all of her native muscles of mastication, namely masseter, temporalis, and medial pterygoid debrided and relying wholly on the VNFs for mastication.

## DISCUSSION

Since the first allogenic face transplant was first performed in 2005,^1^ over 40 cases have been performed worldwide and this currently serves as an exemplar for restoration of both form and function in severely disfigured individuals (eg, following gunshot injuries).^8^ Face transplants for the first time allowed us to restore both function and form in the aesthetic sense. However, with the longer follow-up accorded more than a decade later, its limitations, namely immunosuppression,^9^ death,^10^ and increasing cost implications,^11^ have put the initial euphoria on hold and forced reconstructive surgeons to reflect on the indications for face transplants more carefully.

Although the results, if successful, are excellent, the question remains as to when to draw the line between face transplants and free tissue transfers, but equally the latter currently serve more as indiscriminate volume fillers, particularly after head and neck surgery. These do not provide function and sometimes, actually impair it due to their weight on the face.^12^ This is where there is a need for greater lateral thinking when using free flap reconstruction to utilize all available resources within this autologous tissue, to achieve maximum benefit out of them, for example, by using a muscle flap to recontour facial defects and using its innervating motor nerve to reanimate the face.

The first step in this direction was taken by Koshima et al in 1997^13^ with the use of split chimeric rectus abdominis free flaps to reanimate both the smile and lower lip depression. This novel concept at the time, a product of lateral thinking and resourcefulness, sparked further innovations in the field of FFMTs across the body.^14,15^ However, most of these ideas were confined to the realm of case reports and series with no real common thread.

In the facial reanimation scenario, it was not until Boahene et al in 2018 described the multi-vectorial gracilis,^6^ which worked on a similar principle to the earlier work by Koshima, that this concept gained a foothold in reconstructive surgery. This represented an advancement of the previously described serratus anterior free flap for facial reanimation, but which tended to be too bulky and heavy for facial restorative surgery.^16^ Similarly, an increasing number of chimeric flaps were being utilized in orofacial reconstruction,^17,18^ primarily as tier II reconstruction, followed by isolated reports of tier III reconstructions, providing surface cover, volume, and facial reanimation.^19^

The fusion of composite^4^ and chimeric flap concepts,^5^ along with super-microsurgery and cranial nerve surgery,^20,21^ gives us the ability to restore function and form using free flap technology, courtesy of being able to utilize minute structures of up to 0.3 mm (eg, terminal branches of VII, to achieve better outcomes at an earlier stage),^22^ before motor end-plate degeneration. Even in chronic facial palsy cases (as shown in case 2), these newer concepts can give rise to increasing permutations and combinations in facial restorative surgery, namely VNFs.

VNFs—an advanced version of chimeric flaps with functional elements (eg, nerves and muscles)—provide the long-awaited bridge between allogenic transplants and conventional free flap surgery. Their advantages over facial transplants include, most importantly, no requirement for immunosuppression and the ability to provide up to tier IV reconstructions based on greater understanding of perforator microanatomy for composite defects of up to half of the face. Postoperative recovery is no different from conventional free flaps and has significantly lower psychological afflictions^23^ and ethical conundrums compared with transplants,^24^ especially those of the face. Although VNFs cannot replace the role for face transplants in near-total or total soft tissue and/or bony facial injuries or defects, they offer a viable alternative for the comparatively slightly less severe facial defect (ie, not just focused on “volume fill,” as with conventional free flaps).

Although some may argue that VNFs are in effect free flap reconstructions, one can equally point out that so is the case with facial transplants. In our opinion, it is imperative to differentiate this particular type of reconstruction from conventional free flaps as VNFs can achieve the blend of function and aesthetics so crucial in the face. The key difference between VNFs and free flaps is the need not to look at tissue as a proverbial “brick” but rather understand that it is a conglomeration of many different subunits, each with its individual blood supply, which can be taken apart at the super-microsurgical level and rebuilt into a different composite of tissue altogether. This requires higher order thinking and meticulous planning, but the basic classification of VNFs as described in this article hold true as a template to approach these difficult problems with.

A limitation of this study is its relatively small number, but equally, it must be said that for one of the largest facial palsy centers in the world to only perform five VNFs over a 5-year period is a testimony to the fact that its indications are very few and confined to very severe cases involving the hemi-face but at this junction, excluding the “T-zone” of the face, namely eyes, nose, and mouth. We foresee this to be the next step in the evolution of VNFs.

## CONCLUSION

VNFs are effective reconstructive tools currently used in tier II and III facial deformities where face transplants would be excessive, but where simple tissue filling with conventional free tissue transfer would be rudimentary, in terms of restoring facial function.

## PATIENT CONSENT

The patients provided written consent for the use of their images.
